# Ginsenoside Absorption Rate and Extent Enhancement of Black Ginseng (CJ EnerG) over Red Ginseng in Healthy Adults

**DOI:** 10.3390/pharmaceutics13040487

**Published:** 2021-04-02

**Authors:** Saebyul Yoo, Bom-I Park, Do-hyun Kim, Sooyoung Lee, Seung-hoon Lee, Wang-Seob Shim, Yong Ki Seo, Kimoon Kang, Kyung-Tae Lee, Sung-Vin Yim, Do Yu Soung, Bo-Hyung Kim

**Affiliations:** 1 Department of Biomedical Science and Technology, Graduate School, Kyung Hee University, Seoul 02447, Korea; sbyoo19@khu.ac.kr (S.Y.); dohyun1204@khu.ac.kr (D.-h.K.); 2 Food Research Institutes, CJ CheilJedang, Suwon 16495, Korea; bi.park@cj.net (B.-I.P.); yk.seo@cj.net (Y.K.S.); kimoon.kang@cj.net (K.K.); 3 Department of Life and Nanopharmaceutical Sciences, Graduate School, Kyung Hee University, Seoul 02447, Korea; ls009@khu.ac.kr (S.L.); ktlee@khu.ac.kr (K.-T.L.); 4 Department of Statistics, lnha University, Incheon 22212, Korea; enql3824@gmail.com; 5 Kyung Hee Drug Analysis Center, College of Pharmacy, Medical Center, Kyung Hee University, Seoul 02447, Korea; wsshimm@khu.ac.kr; 6 Department of Pharmaceutical Biochemistry, College of Pharmacy, Kyung Hee University, Seoul 02447, Korea; 7 Department of Clinical Pharmacology and Therapeutics, Kyung Hee University Medical Center, Seoul 02447, Korea; ysvin@khu.ac.kr; 8 East-West Medical Research Institute, Kyung Hee University, Seoul 02447, Korea

**Keywords:** black ginseng, ginsenosides, pharmacokinetics

## Abstract

Red ginseng (RG) and black ginseng (BG, CJ EnerG) were prepared from fresh ginseng using one and nine cycles of steaming and drying, respectively. This process reduces the molecular weight (MW) of ginsenoside-active compounds in ginseng by removing sugar moieties from their dammaranes. We compared the pharmacokinetic characteristics of ginsenosides between BG comprising mainly low-MW ginsenosides (Rg3, Rg5, Rk1, and Rh1) and RG that predominantly contains high-MW ginsenosides (Rb1, Rb2, Rc, Rd, Re, and Rg1). The safety profiles and tolerability were also studied using a randomized, double-blind, single-dose, crossover clinical trial. A combination of Rb1, Rg1, and Rg3, well-known representative and functional RG components, exhibited a 1 h faster absorption rate (T_max_) and 58% higher exposure (24 h area under the concentration–time curve, AUC_24_) in BG than in RG. Furthermore, the combination of Rg3, Rg5, and Rk1, the major and most efficient components in BG, displayed 824% higher absorption (AUC_24_) in BG than in RG. The total ginsenoside showed a 5 h rapid intestinal absorption (T_max_) and 79% greater systemic exposure (AUC_24_) in BG than in RG. No clinically significant findings were observed in terms of safety or tolerability. Thus, BG extract was more effective than RG extract.

## 1. Introduction

**Figure 1:**
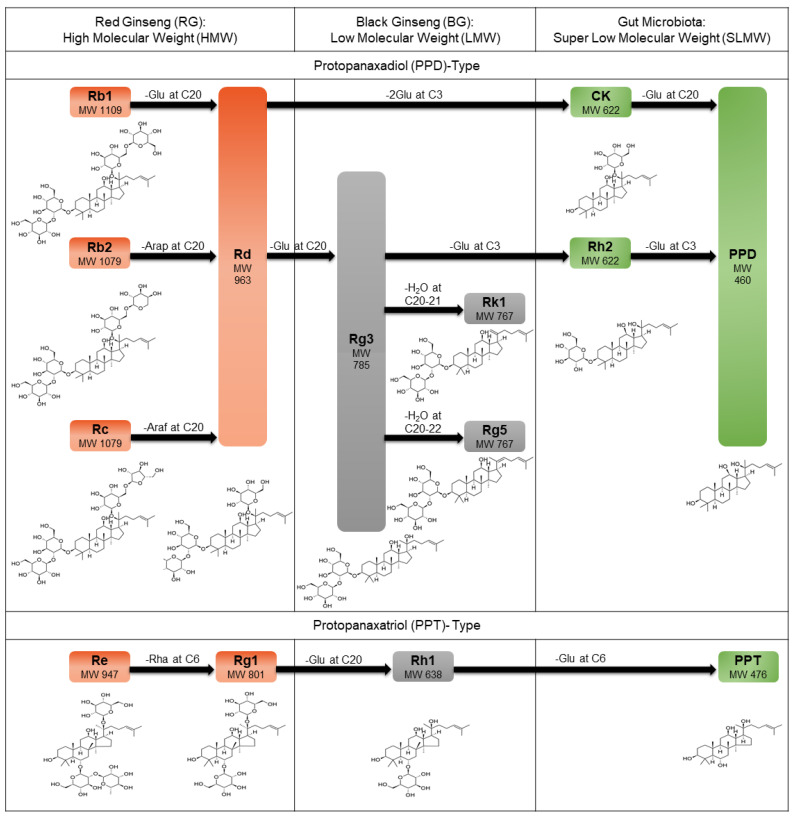
Multiple steaming cycles and gut microbial activity reduce the molecular weight (MW) of ginsenosides in ginseng. Ginsenosides are tetracyclic triterpene (dammarane)-type saponins and are divided into protopanaxadiol (PPD) type (e.g., Rb1, Rb2, Rc, Rd, Rg3, Rk1, Rg5, CK, Rh2, and PPD) and protopanaxatriol (PPT) type (e.g., Re, Rg1, Rh1, and PPT). Based on their size, ginsenosides are divided into three groups: (1) high molecular weight (HMW) (>800) ginsenosides found in red ginseng (RG) such as Rb1 (MW: 1109), Rb2 (MW: 1079), Rc (MW: 1079), Rd (MW: 1079), Re (MW: 947), and Rg1 (MW: 801); (2) low MW (LMW) (>637, <800) ginsenosides in black ginseng (BG) such as Rg3 (MW: 785), Rk1 (MW: 767), Rg5 (MW: 767), and Rh1 (MW: 638); and (3) super low MW (SLMW) (<623) ginsenosides such as CK (MW: 622), Rh2 (MW: 622), PPD (MW: 460), and PPT (MW: 476). In PPD type, Rb1, Rb2, Rc, and Rd are deglycosylated at C20 on dammarane to generate Rg3 using heat. Rk1, with a double bond at C20–21, and Rg5, with a double bond at C20–22, are produced from Rg3 through heat-mediated dehydration. Rd and Rg3 are further transformed into CK and Rh2, respectively, and eventually to PPD by gut flora. Re and Rg1 are PPT types, which are used to generate Rh1 by sequentially deleting rhamnose at C6 and glucose at C20. The outer glucose residue of Rh1 at C6 is removed by microbial enzymes to form PPT. Glu: glucose; Arap: arabinose (pyranose form); Araf: arabinose (furanose form); Rha: rhamnose.

Ginseng (*Panax ginseng* C. A. Meyer) is a widely used traditional medicinal plant not only in Asian countries, such as Korea, China, and Japan, but also in the United States and Europe. Ginseng possesses several pharmacological activities, including anti-inflammatory, anti-diabetic, anti-tumor, immune-regulatory, anti-oxidation, and anti-fatigue properties. These activities are attributed to the pharmacological action of dammarane-type saponins, known as ginsenosides, which are the active components of ginseng [1,2,3,4]. Gin senosides are classified as protopanaxadiol (PPD) and protopanaxatriol (PPT) types. Examples of PPD-type ginsenosides are Rb1, Rb2, Rc, Rd, Rg3, Rk1, Rg5, Rh2, compound K (CK), and PPD, whereas PPT-type ginsenosides include Re, Rg1, Rh1, and PPT (Figure 1).

Ginseng is categorized into fresh ginseng (FG), red ginseng (RG), and black ginseng (BG) based on its processing method. Fresh ginseng is undried ginseng, whereas RG is synthesized by steaming and drying FG. Black ginseng is produced by nine cycles of FG steaming and drying. The ratio of components of ginsenosides changes with an increase in the number of cycles of steaming and drying. For example, the increase in Rg3, Rg5, and Rk1 components and the decrease in Rb1 and Rg1 components are a result of steaming and drying processes. Therefore, BG has a higher amount of Rg3, Rg5, and Rk1 and a lower amount of Rb1 and Rg1, than RG. In addition, BG has a higher amount of ginsenosides with a molecular weight (MW) <800 than RG because of the molecular modification during steaming and drying [5].

Rg3, Rg5, and Rk1 have several pharmacological activities including anti-tumor effects and immune system modulation, whereas Rb1 and Rg1 are known for their cognitive enhancement and cardioprotective effects [6,7,8,9,10,11,12,13]. In addition, compared with RG, BG is more effective in controlling the immune system to protect the lungs against influenza virus (H1N1) infection in mice. It also exerts significant anti-inflammatory and anti-nociceptive effects both in vivo and in vitro [14,15]. Furthermore, BG showed a better cognitive-enhancing effect than RG in old mice via regulation of the cholinergic system [16].

Several in vitro and in vivo studies have reported greater pharmacological effects of BG than RG. However, the effect of ginsenoside composition on the pharmacokinetics (PK) of different types of ginsenosides after administration of BG and RG to the same individual has not been reported. Thus, to compare and evaluate the PK properties of 14 ginsenosides, BG (CJ EnerG) extract was administered to the test group and RG extract was administered to the control group. In addition, we performed a comparative evaluation of the safety and tolerability of BG and RG.

## 2. Materials and Methods

### 2.1. Materials

**Figure 2:**
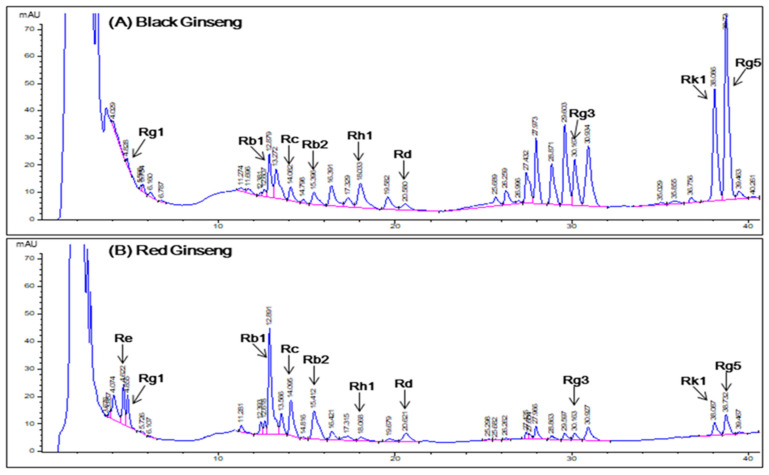
Chromatograms obtained from high-performance liquid chromatography analysis of ginsenosides in (**A**) black ginseng and (**B**) red ginseng extracts. Detection at a wavelength of 203 nm.

Black ginseng (CJ EnerG) and RG extracts were obtained from the CJ CheilJedang Corporation (Suwon, Korea). Black ginseng and RG were extracted by adding a solution of ethanol and water to a heat reflux extraction system. We used 9 g of BG and RG extracts as a single dose. Quantification of ginsenosides in each BG and RG extract was performed using high-performance liquid chromatography (HPLC) with a diode-array detector (Agilent 1260, Santa Clara, CA, USA) equipped with a Venusil XBP C18 column (4.5 × 250 mm, ID 5 µm). The mobile phases were acetonitrile (A) and water (B). The injection volume was 5 µL with a gradient as follows: 0–4.51 min, 30% A; 4.51–6 min, 30–40% A; 6–18 min, 40% A; 18–25 min 40–55% A; 25–30 min, 55% A; 30–45 min, 55–90% A; 45–50 min, 90–100% A; 50–52 min, 100–30% A. Column oven temperature was 50 °C (Figure 2). The ginsenoside content (mg/9 g) of the BG extract was Rb1, 17.91; Rb2, 6.39; Rc, 4.95; Rd, 6.93; Rg3, 38.79; Rg5, 72.81; Rk1, 33.48; Rg1, 1.89; and Rh1, 12.33. The ginsenoside contents (mg/9 g) of RG extract were Rb1, 48.78; Rb2, 19.62; Rc, 19.35; Rd, 8.1; Rg3, 4.59; Rg5, 6.48; Rk1, 0.99; Re, 26.1; Rg1, 19.08; and Rh1, 0.99.

### 2.2. Study Participants

All participants were healthy Korean male volunteers aged 19 to 45 years. All participants signed an informed consent form before screening. Physical examination, vital signs, 12-lead electrocardiography, clinical laboratory tests (hematology, chemistry, and urinalysis), and serology (hepatitis B virus surface antigen, hepatitis C virus antibody, and anti-HIV antibody) revealed no clinically significant abnormalities.

### 2.3. Study Design

This was a randomized, double-blind, single-dose, crossover study at the Clinical Trial Center of Kyung Hee University Hospital (Seoul, Korea). The subjects were randomly assigned to two sequence groups. The subjects in the first sequence group were administered BG extract (9 g) during the first hospitalization period and subsequently administered RG extract (9 g) during the second hospitalization period. The subjects in the second sequence group were administered RG extract during the first hospitalization period and subsequently administered BG extract during the second hospitalization period. Each hospitalization period was separated by a 14 d washout period. Blood samples were collected in heparinized tubes before administration (0 h) and at 0.5, 1, 2, 3, 4, 5, 6, 7, 8, 10, 12, 14, 24, 30, and 48 h after the administration of BG or RG extract. Plasma was collected by centrifugation at 1800× *g* for 10 min and stored at −70 °C until analysis.

All study procedures were conducted in accordance with the principles of the Declaration of Helsinki and Korean Good Clinical Practice guidelines. The protocol and informed consent forms were approved on 11, November 2019 by the Institutional Review Board (IRB number: 2019-10-023-002) of Kyung Hee University Hospital (Seoul, Korea).

### 2.4. Bioanalytical Methods

Rb1, Rb2, Rc, Rd, Re, Rg1, Rg3 (20S), Rg5, and Rk1 were separated as described previously [17]. The Agilent Infinity 1260 (Agilent Technologies, Santa Clara, CA, USA) and Agilent 6470 Triple Quadrupole Mass Spectrometer (Agilent Technologies, Santa Clara, USA), with the electrospray ionization source in positive and negative ion modes, were used. Data were analyzed and processed using the Mass Hunter Acquisition Software (Version B.08.00; Agilent Technologies, Santa Clara, USA). Rh1 (20S), Rh2 (20S), CK, PPD (20S), and PPT (20S) were separated using the Shimadzu Nexera X2 (Shimadzu, Kyoto, Japan) and the Sciex API 4000 Triple Quadrupole Mass Spectrometer (SCIEX, Framingham, MA, USA) with an electrospray ionization source in negative ion mode. Data were acquired and processed using the Analyst 1.62 program (version 1.62, SCIEX, Framingham, USA). The final concentrations of the calibration standard were 0.5, 1, 2, 5, 10, 25, and 50 ng/mL, and those of the quality control (QC) samples were 1.5, 7.5, and 40 ng/mL. Human plasma was removed at −80 °C and dissolved at 20 to 25 °C. Verification of analytical methods was validated for selectivity, linearity, accuracy, precision, recovery, matrix effect and stability according to the guidelines of the bioanalytical method, Korean Ministry of Food and Drug Safety (MFDS) guidelines.

### 2.5. Pharmacokinetics

Pharmacokinetic parameters were obtained using non-compartmental analysis methods using the Phoenix WinNonlin 8.1 (Certara USA Inc., Princeton, NJ, USA). The maximum concentration (C_max_) and time to reach C_max_ (T_max_) were directly acquired from individual datasets of the concentration–time profiles. The area under the concentration–time curve (AUC) was calculated using the linear-up/log-down trapezoidal rule for the non-compartmental analysis method. The AUC from 0 to 24 h (AUC_24_) was determined from concentrations where the values below the lower limit of quantification (LLOQ) were substituted by zero, and AUC from 0 to the actual time point relevant to the last concentration above the LLOQ (AUC_last_) was calculated from the dataset of actual concentrations.

### 2.6. Safety and Tolerability Assessment

Safety and tolerability were assessed by studying the adverse events reported by the subjects throughout the duration of the clinical study and serial measurements of blood pressure and body temperature, which were monitored before administration and at 2, 4, 8, 14, 24, 30, and 48 h after the administration of BG or RG extract. Physical examination and clinical laboratory tests were conducted before administration of BG or RG and discharge.

To assess the safety profile, the severity, duration, outcome, and relationship of adverse events were evaluated.

### 2.7. Statistical Analysis

Descriptive statistics were applied to summarize all demographic characteristics, PK parameters, and safety profiles. The PK parameters between BG and RG were compared using log-transformed C_max_ and AUC values_._ The AUC_24_ values were analyzed using a linear mixed-effect model that incorporated both fixed-effect parameters for sequence, hospitalization period and treatment, and a random-effect parameter for the subject. In these mixed-model procedures, the treatment differences between BG and RG were back-transformed to obtain the ratios of geometric least-squares means and the corresponding statistics, which were used to calculate C_max_ and AUC_24_. T_max_ was compared between BG and RG using Wilcoxon’s signed-rank test.

## 3. Results

### 3.1. Subjects

**Figure 3:**
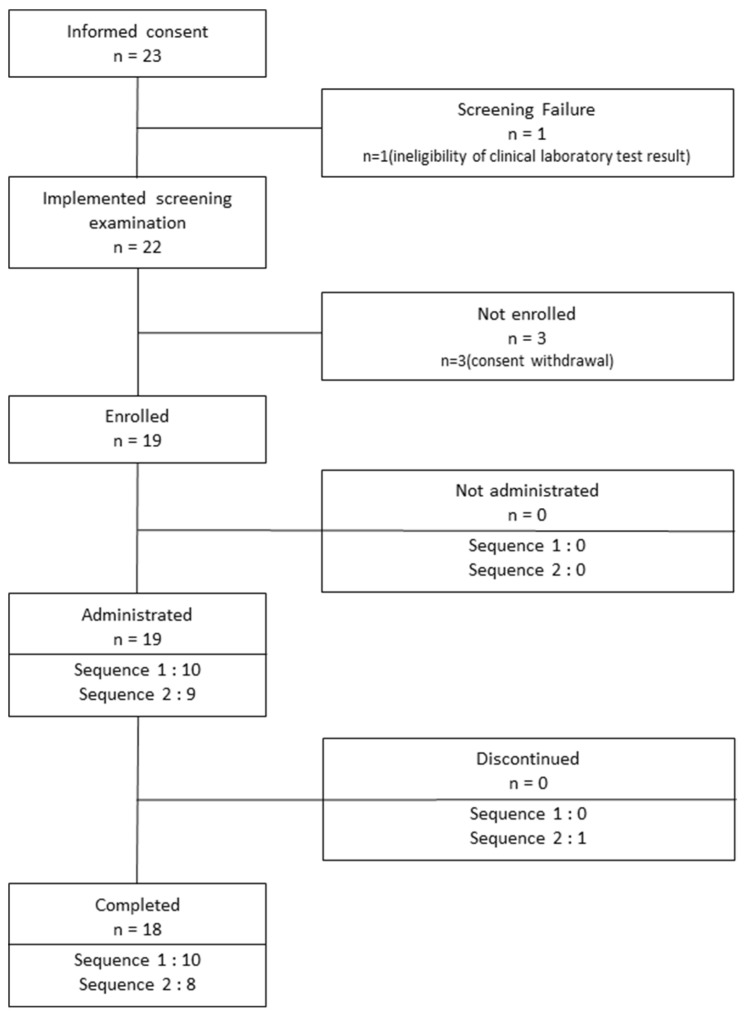
Flowchart showing subject disposition.

Twenty-three healthy Korean male volunteers were included in this study. One of the volunteers was found to be ineligible for the study because of high serum levels of alanine aminotransferase (AST) and aspartate aminotransferase (ALT). Three volunteers withdrew their consent for participation. The remaining 19 subjects were enrolled in the study. However, one subject dropped out of the study because he took medication for diarrhea during the wash-out period after the first hospitalization period. Consequently, 18 subjects completed the clinical study (Figure 3). The mean ± standard deviation of age, height, and weight of the 18 enrolled subjects were 26.72 ± 3.34 years, 175.71 ± 5.04 cm, and 73.57 ± 6.83 kg, respectively. No significant differences in age, height, or weight were observed between sequence groups 1 and 2.

### 3.2. Validation of the Bioanalytical Method

The selectivity was determined by analyzing six different blank human plasma samples and the combined plasma to evaluate the presence of endogenous materials. These samples were subjected to the pretreatment procedure described in Section 2.4. The results indicated that the analytes and internal standards were observed to be free of interference from each other. The LLOQ (0.5 ng/mL) was determined with signal-to-noise ratios >10. The precision and accuracy of several LLOQ samples are ensured and are applicable to bioavailability studies. The calibration curves were prepared for ginsenoside concentrations of 0.5–50 ng/mL and linearities were determined using linear regression (1/*x*^2^ weighting factor). The correlation coefficients were above 0.99 for all curves, which is an indicator of the linearity and repeatability being within the analysis range. The intra-day precision range ranged from 0.8% to 19.5%, and the accuracy ranged from 84.5% to 114.82%. The inter-day precision range was 1.4% to 19.3%, and the accuracy ranged from 89.4% to 107.9%. The results were within the 15% range (20% at LLOQ) of precision (%) and accuracy (%) required by the MFDS guidelines.

Recovery and matrix effects were determined by assessing the ion enhancement or suppression caused by the matrix. Recovery was determined at three QC concentrations by comparing the analytical peak area of the QC sample and the peak area injected at corresponding concentrations with the blank plasma after extraction. The matrix effect was determined by comparing the peak area of the QC concentrations of the standard solution and the corresponding areas of the same concentration of the analyte injected into the residue extracted from the blank plasma.

### 3.3. Pharmacokinetics

#### 3.3.1. Pharmacokinetic Parameters of Individual Ginsenosides

The PK parameters of the individual ginsenosides obtained from the plasma concentration–time profiles are shown in Table 1. The C_max_ of all ginsenosides is described as the mean ± standard deviation (standard error) (ng/mL). The T_max_ of all ginsenosides is described as the median (min, max) in hours. In addition, the AUC_24_ and AUC_last_ of all ginsenosides are represented as the mean ± standard deviation (standard error) in ng h/mL. All PK parameters of ginsenosides were statistically compared between the BG and RG extract groups.

**Figure 4:**
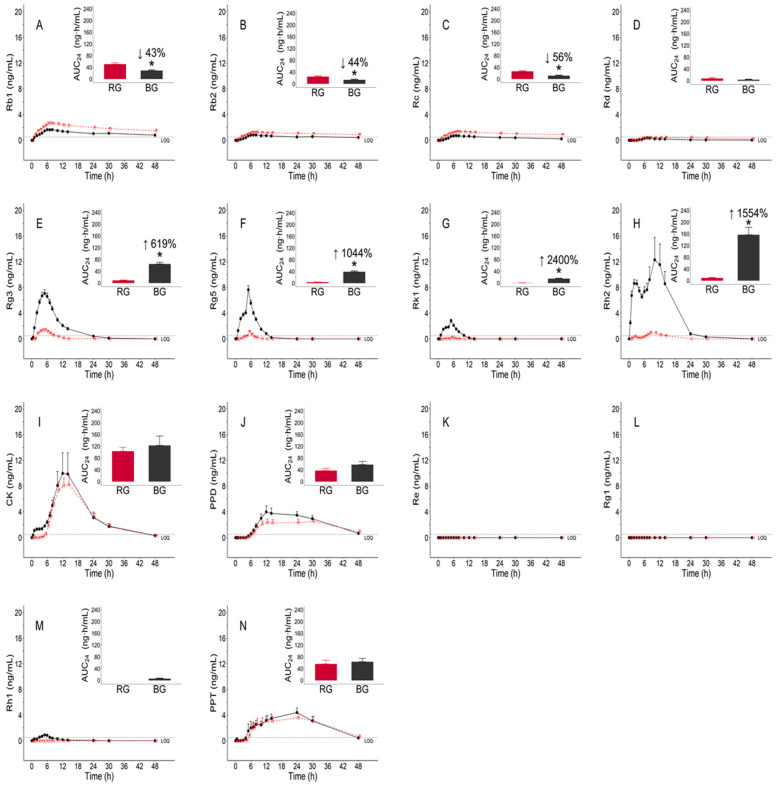
Mean (± standard error (SE)) plasma concentration–time profiles of ginsenosides after administration of a single dose of 9 g of red ginseng (RG) (red empty symbol and dashed line) and black ginseng (BG) (black filled symbol and solid line) extract. The inserted bar plot showing the mean (±SE) for the 0 to 24 h area under the concentration–time curve (AUC_24_) of RG (red color) and BG (black color) extract: (**A**) Rb1; (**B**) Rb2; (**C**) Rc; (**D**) Rd; (**E**) Rg3; (**F**) Rg5; (**G**) Rk1; (**H**) Rh2; (**I**) CK; (**J**) PPD; (**K**) Re; (**L**) Rg1; (**M**) Rh1; (**N**) PPT. * Statistically significant at *p* < 0.05.

Regarding Rb1, Rb2, Rc, and Rd, all PK parameters of C_max_, AUC_24_, and AUC_last_ for the RG extract were higher than those for the BG extract. All median values of T_max_ for the RG extract were similar to those for the BG extract. The concentration versus time profiles of Rb1, Rb2, Rc, and Rd are shown in Figure 4A–D. The C_max_, AUC_24_, and AUC_last_ of Rb1, Rb2, and Rc were significantly different between RG and BG (*p* < 0.05). The mean AUC_24_ values of Rb1, Rb2, and Rc in the BG group were 43%, 44%, and 56% lower, respectively, than those in the RG group.

Regarding Rg3, Rg5, Rk1, Rh2, CK, and PPD, the C_max_, AUC_24_, and AUC_last_ for the BG extract were higher than those for the RG extract, except for the mean C_max_ of CK. All median values of T_max_ for the RG extract were the same as those for the BG extract, except for Rg3. The PK profiles of Rg3, Rg5, Rk1, Rh2, CK, and PPD are shown in Figure 4E–J. The C_max_, AUC_24_, and AUC_last_ of Rg3, Rg5, Rk1, and Rh2 were significantly different between RG and BG (*p* < 0.05). The mean AUC_24_ values of Rg3, Rg5, Rk1, and Rh2 were 619%, 1044%, 2400%, and 1554% higher, respectively, in the BG group than in the RG group.

All Re concentrations were below the LLOQ (Figure 4K). In addition, all concentrations of Rg1, except for one, were below the LLOQ (Figure 4L). Therefore, the parameters Re and Rg1 could not be evaluated. Because none of the RG concentrations of Rh1 were detected or were below the LLOQ, we could not evaluate the C_max_, T_max,_ and AUC_last_ of RG. In addition, as the AUC_24_ value of RG was zero, the log-transformed AUC_24_ could not be defined, and RG and BG groups could not be statistically compared.

#### 3.3.2. Pharmacokinetic Parameters of Functional Ginsenosides Group

**Figure 5:**
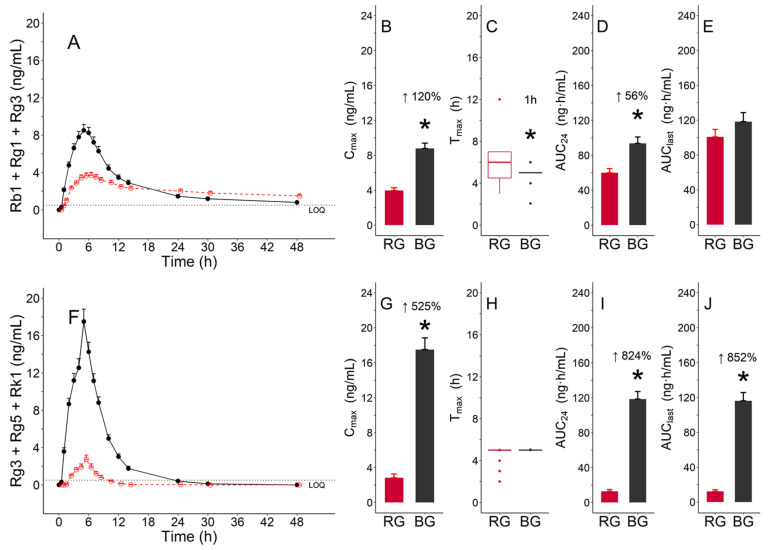
Pharmacokinetic characteristics of functional ginsenoside groups after the administration of a single dose of red ginseng (RG) (red color) and black ginseng (BG) (black color) extracts: (**A**–**E**) Functional ginsenoside group with Rb1, Rg1 and Rg3; (**F**–**J**) functional ginsenoside group with Rg3, Rg5, and Rk1; (**A**,**F**) mean (± standard error (SE)) plasma concentration–time profile of group 1; (**B**,**G**) mean (±SE) for C_max_; (**C**,**H**) median (inter-quartile range (IQR)) boxes for T_max_ (lines for 1.5 times the IQR and dots for outliers); (**D**,**I**) mean (±SE) for area under the concentration–time curve AUC from 0 to 24 h (AUC_24_); (**E**,**J**) mean (±SE) for AUC from 0 to the actual time point relevant to the last concentration above the lower limit of quantification (LLOQ) (AUC_last_). * Statistically significant at *p* < 0.05.

There were two functional ginsenoside groups; one group consisted of Rb1, Rg1, and Rg3, and the other group consisted of Rg3, Rg5, and Rk1. These groups exhibited a significant difference in C_max_ and AUC_24_ between BG and RG (*p* < 0.05). The C_max_ (mean ± standard deviation, ng/mL) values for the group with Rb1, Rg1, and Rg3 were 4.0 ± 1.4 and 8.8 ± 2.6 for RG and BG, respectively; the AUC_24_ (mean ± standard deviation, ng·h/mL) values were 60.0 ± 20.9 and 93.6 ± 31.2, respectively, for RG and BG (Table 2, Figure 5B,D). The T_max_ (median (min, max), hours) values were 6.0 (3.0, 12.0) and 5.0 (2.1, 6.0) for RG and BG, respectively (Table 2). A significant difference was observed between the two treatments (Figure 4C). The values for C_max_ (mean ± standard deviation, ng/mL) for the other functional ginsenoside group with Rg3, Rg5 and Rk1 were 2.8 ± 1.9 and 17.5 ± 5.7 for RG and BG, respectively; the AUC_24_ (mean ± standard deviation, ng·h/mL) values were 12.8 ± 8.7 and 118.3 ± 37.0 for RG and BG, respectively (Table 2, Figure 5G,I). In addition, this group showed a significant difference in AUC_last_ between BG and RG (*p* < 0.05) (Figure 5J).

#### 3.3.3. Pharmacokinetic Parameters of Total Ginsenosides

**Figure 6:**
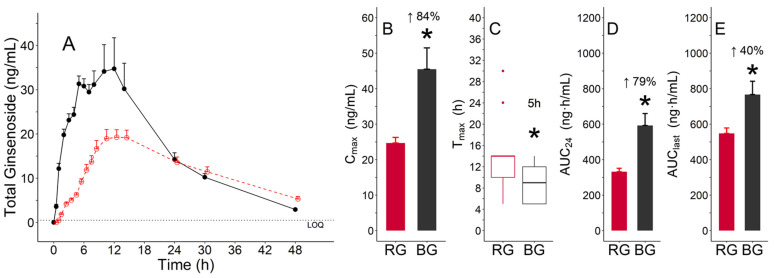
Pharmacokinetic characteristics of total ginsenosides after the administration of a single dose of red ginseng (RG) (red color) and black ginseng (BG) (black color) extracts: (**A**) mean (±standard error (SE)) plasma concentration–time profile; (**B**) mean (±SE) for C_max_; (**C**) median (inter-quartile range (IQR)) boxes for T_max_ (lines for 1.5 times the IQR and dots for outliers); (**D**) mean (±SE) for area under the concentration–time curve from 0 to 24 h (AUC_24_); (**E**) mean (±SE) for AUC from 0 to the actual point in time with respect to the last concentration above the lower limit of quantification (LLOQ) (AUC_last_). * Statistically significant at *p* < 0.05.

The mean concentration–time profiles of total ginsenosides showed that the mean concentrations of BG from 0 to 24 h were higher than those of RG (Figure 6A). The differences in all parameters were significant between BG and RG (*p* < 0.05). The C_max_ (mean ± standard deviation, ng/mL) values of RG and BG were 24.7 ± 6.9 and 45.5 ± 25.4, respectively (Table 3, Figure 6B). The T_max_ (median (min, max), h) values of RG and BG were 14.0 (5.0, 30.0) and 9.0 (5.0, 14.0), respectively (Table 3, Figure 6C). The AUC_24_ (mean ± standard deviation, ng·h/mL) values of RG and BG were 331.6 ± 85.3 and 592.4 ± 285.9, respectively, and AUC_last_ values of RG and BG were 547.6 ± 134.3 and 767.0 ± 317.4, respectively (Table 3). The AUC_24_ and AUC_last_ (79% and 40%, respectively) of BG were significantly higher than those of RG (Figure 6D,E).

### 3.4. Safety and Tolerability

We report one treatment-emergent adverse event (TEAE), i.e., diarrhea, following the administration of RG extract. The TEAE was mild in intensity and was not related to RG extract administration. The TEAE was completely resolved after the medications were administered to reduce symptoms. No serious adverse events were observed. No changes were observed in the clinical laboratory test findings, blood pressure, body temperature, or upon physical examination.

## 4. Discussion

In this study, the contents of Rg3, Rg5, Rk1, and Rh1 in the BG extract were higher than those in the RG extract. The RG extract had a higher content of Rb1, Rb2, Rc, Rd, Re, and Rg1 than the BG extract. The systemic exposure of ginsenosides was studied by calculating T_max_, C_max_, and AUC and by performing PK analysis. Two types of AUCs were calculated for each subject. AUC_last_ shows ginsenoside exposure from 0 to the time point with the last measurable concentration above LLOQ, and AUC_24_ shows systemic exposure during a 24 h period. A comparison of the C_max_ and AUC of the RG and BG groups revealed that the systemic exposure of Rg3, Rg5, Rk1, and Rh2 in the BG group was higher than that in the RG group. Furthermore, the PK comparisons of total ginsenosides between both groups showed a greater systemic exposure and faster absorption rate after administration of BG extract than after administration of RG extract.

The functional ingredients of BG have not yet been defined. Rb1, Rg1, and Rg3 are used as functional ingredients of RG based on the Health Functional Food Code (No. 2020-92), MFDS [18]. Based on the functional ingredients of RG, daily administration of 2.4–80 mg/g Rb1, Rg1, and Rg3 contained in RG extract had several health benefits such as enhanced immunity, relief from fatigue, improved blood flow via inhibition of platelet aggregation, memory improvement, antioxidation effects, and positive effects on menopausal female health [18]. Therefore, their PK characteristics in each group were compared and analyzed after administration of RG and BG extracts. The Rb1, Rg1, and Rg3 combination of BG was absorbed 1 h faster than that of RG. In addition, the C_max_ and AUC_24_ of the combination of Rb1, Rg1, and Rg3 were 120% and 56% higher, respectively, in BG than in RG (Figure 5). Thus, the combination of Rb1, Rg1, and Rg3 in BG was absorbed at a faster rate and to a greater extent than that in RG. These results suggest that BG exerted higher beneficial pharmacological effects than RG.

The Rg3, Rg5, and Rk1 content was higher than that of other ginsenosides in the BG extract. In addition, the systemic exposures of these ginsenosides, that is, C_max_ and AUC, were higher than those of other ginsenosides after receiving BG extract. Therefore, Rg3, Rg5, and Rk1 can be considered the functional ingredients of BG. Based on this finding, the concentrations of these ingredients were summed for each subject to calculate the PK parameters for several BG ginsenosides. Pharmacokinetic analysis of the combination of Rg3, Rg5, and Rk1 showed that C_max_, AUC_24_, and AUC_last_ of BG were 525%, 824%, and 852% higher, respectively, than those of RG (Figure 5). In addition, numerous studies have shown the anti-inflammatory effects of Rg3, Rg5, and Rk1. For example, Rg3 attenuated the increase in pro-inflammatory markers, such as tumor necrosis factor (TNF)-α, interleukin (IL)-1β, and cyclooxygenase (COX)-2, in the hippocampus. Rg5 suppressed the production of TNF-α and IL-1β and reduced the expression of nuclear factor (NF)-κB p65 and COX-2 in cisplatin-treated mice with renal inflammation. Similarly, Rk1 significantly inhibited the overproduction of TNF-α and IL-1β and reduced the expression of inducible nitric oxide synthase (iNOS) and COX-2 in mouse liver tissues [19,20,21]. Furthermore, several in vitro and in vivo studies have shown that BG extract reduces inflammation through an immune-modulation mechanism [14,15]. Therefore, the pharmacological effects of BG extract were higher than those reported after receiving the same amount of RG extract.

The content of Rb1, Rb2, and Rc was higher than that of other ginsenosides in the RG extract, and similar to the content of Rg3, Rg5, and Rk1 in the BG extract. The median T_max_ values of Rg3, Rg5, and Rk1 were 4 to 5 h, and those of Rb1, Rb2, and Rc were 7–8 h. These findings suggest that the absorption rates of Rb1, Rb2, and Rc were relatively lower than those of Rg3, Rg5, and Rk1. Rb1, Rb2, and Rc are polar ginsenosides because of the presence of sugar molecules at the C20 position, as opposed to Rg3, Rg5, and Rk1, which do not contain sugar molecules at this position. This polarity can slow the permeation rate through the intestinal membrane, thereby disturbing the absorption of Rb1, Rb2, and Rc. In addition, the sum of AUC_last_ of Rg3, Rg5, and Rk1 after receiving the BG extract and that of AUC_last_ of Rb1, Rb2, and Rc after receiving the RG extract, were 114.7 and 192.0 ng·h/mL, respectively. Based on the AUC_last_ and median T_max_ values, we concluded that ginsenosides Rb1, Rb2, and Rc were absorbed slowly over a longer duration than Rg3, Rg5, and Rk1.

Among the PPT-type ginsenosides, the content of Re and Rg1 in the RG extract was higher than that in the BG extract; however, Re and Rg1 were not absorbed after administration of RG and BG extracts. According to a previous study, neither Re nor Rg1 was absorbed in subjects administered RG containing these ginsenosides daily for 2 weeks [22]. In the current study, Rh1 was not absorbed after receiving RG extract; however, Rh1 concentrations were detected after the administration of BG extract. The BG extract (9 g) contained 12.33 mg of Rh1 with a low MW, and the RG extract (9 g) contained 0.99 mg of Rh1. The higher Rh1 content in the BG extract resulted in systemic exposure to Rh1 only after the administration of BG extract. Re and Rg1 have sugar molecules at the C6 and C20 positions, respectively. The sugar molecules were removed during the formation of Rh1, making the absorption of Rh1 easier than that of Re and Rg1. Unlike Re, Rg1, and Rh1, the systemic exposure to PPT—the end product of metabolism—was high in both RG and BG groups. We believe that almost all the absorbed ginsenosides, Re, Rg1, and Rh1, were metabolized into PPT through the gastrointestinal tract and liver. Therefore, anti-diabetic and kidney-protective effects and protective effects against cerebral ischemia reported previously could be associated with the effects of the metabolites Rh1 and PPT, rather than the direct pharmacological activities of Re and Rg1 [23,24,25,26].

CK, Rh2, PPD, and PPT are metabolized from other ginsenosides by the gut microbiota [27,28,29]. The mean AUC_24_/AUC_last_ values of CK in the RG and BG groups were 103.5/136.2 and 123.0/147.5 ng·h/mL, respectively. There was no significant difference between RG and BG, although systemic exposures were slightly higher in the BG than in the RG group. The PK difference in CK between both groups could be ascribed to the metabolism of Rd into CK. The Rd contents of RG and BG extracts were 8.10 and 6.93 mg/9 g, respectively, and the mean AUC_24_/AUC_last_ values of RG and BG were 7.8/18.2 and 3.6/9.6 ng·h/mL. The mean AUC values of Rd were considerably lower than those of CK after administration of RG and BG extracts. Therefore, Rd is absorbed less and metabolized rapidly to CK. The mean AUC_24_/AUC_last_ values of Rh2 in RG and BG were 9.5/8.2 and 157.1/152.1 ng·h/mL, respectively, suggesting that the systemic exposure of Rh2 after the administration of BG extract was significantly higher than that after BG administration. The content of Rg3, a precursor of Rh2, in BG extract was higher than that in RG extract; the content of Rg3 was 38.79 mg/9 g in BG extract and 4.59 mg/9 g in RG extract, and accounted for approximately 20% and 3%, respectively, of the total amount of the 14 ginsenosides in the BG and RG extracts. In addition, systemic exposure to Rg3 after administration of BG was significantly higher than that after RG administration. Thus, it can be speculated that a large amount of Rg3 after absorption is metabolized into Rh2. In the case of PPD, no statistically significant difference in systemic exposure between the two groups was observed. The mean AUC_24_/AUC_last_ values of PPD in BG and RG were 57.8/107.3 and 37.5/80.0 ng·h/mL, respectively. These minimal PK differences in PPD between the two groups could be explained by the PK of PPD precursors. Systemic exposure to CK was similar in both the groups. However, the expression of Rh2 was significantly higher in the BG group than in the RG group. Rh2 was more likely to be absorbed as such rather than undergoing biotransformation to PPD. Consequently, systemic exposure to PPD could be affected by the metabolism of CK compared to that of Rh2. Therefore, the efficacy of CK did not result in a significant difference between both the groups. However, Rh2, which has anti-inflammatory and anti-cancer effects, is expected to exert a remarkable effect in BG compared to RG [30,31,32].

The PK parameters for total ginsenosides were assessed after the concentrations of total ginsenosides were obtained by summing the concentrations of individual ginsenosides for each subject. The T_max_ of the total ginsenoside content of BG was 5 h faster than that of RG. The different absorption rates of total ginsenosides could be caused by the differences in Rg3, Rg5, and Rk1 content between the BG and RG extracts; the content of Rg3, Rg5, and Rk1 was approximately 12 times higher in BG (145.08 mg/9 g) than in RG (12.06 mg/9 g). The C_max_, AUC_24_, and AUC_last_ of BG were 84%, 79%, and 40% higher than those of RG, respectively, indicating that the overall systemic exposure of total ginsenosides for BG was significantly higher than that of RG (Figure 6). These findings could result from the differences in total ginsenoside content; the total ginsenoside content in the BG extract (195.48 mg/9 g) was approximately 1.3 times higher than that in the RG extract (154.08 mg/9 g).

All subjects were administered a single dose of BG and RG extracts for a certain wash-out period. Despite the controlled clinical study, the absorbed concentration of ginsenosides can differ among subjects owing to individual gut microbiota, metabolic capability, and demographic characteristics. Thus, RG and BG are frequently taken several times per day over multiple days. The results of the current study could form the basis for studying the PK of multiple doses. A multi-dose study to evaluate the PK of ginsenosides via absorption, metabolism, and excretion at a steady state can provide information on appropriate BG dosage regimens. In addition, the PK results of this study can be used to interpret the efficacy of further studies on BG and contribute to defining its functional ingredients.

## 5. Conclusions

The PK characteristics of the 14 ginsenosides were analyzed by administering a single dose of RG and BG. The PK results suggested that the systemic exposures (AUC_24_) and T_max_ of total ginsenosides were 79% higher and 5 h earlier in the BG extract than in the RG extract. In particular, the systemic exposure of functional ingredients comprising Rb1, Rg1, and Rg3 in RG, as well as ingredients primarily comprising Rg3, Rk1, and Rg5 in BG, were higher in the BG extract than in the RG extract. These findings indicate that BG is likely to be more effective than RG. In addition, no adverse events related to RG or BG and no clinically significant findings in the assessment of safety and tolerability were found.

## Figures and Tables

**Table 1:** Pharmacokinetic (PK) parameters of individual ginsenosides in healthy male volunteers after a cross-over administration of black ginseng (BG) and red ginseng (RG).

Ginsenosides	TRT (N ^1^)	PK Parameters			
		C_max_ (ng/mL)	T_max_ (h)	AUC_24_ (ng·h/mL)	AUC_last_ (ng·h/mL)
Rb1	RG (19)	2.8 ± 1.0 (0.2)	7.0 (4.0, 14.0)	51.0 ± 18.9 (4.3)	91.7 ± 35.4 (8.1)
	BG (18)	1.8 ± 0.7 (0.2) *	7.0 (5.0, 12.0)	29.1 ± 12.0 (2.8) *	51.5 ± 23.4 (5.5) *
Rb2	RG (18)	1.5 ± 0.5 (0.1)	7.5 (5.0, 48.0)	24.6 ± 11.5 (2.6)	50.8 ± 21.6 (5.1)
	BG (15)	1.1 ± 0.4 (0.1) *	7.0 (5.0, 12.0)	13.7 ± 9.9 (2.3) *	30.9 ± 17.7 (4.6) *
Rc	RG (19)	1.5 ± 0.5 (0.1)	7.0 (6.0, 14.0)	26.4 ± 10.1 (2.3)	49.5 ± 20.9 (4.8)
	BG (15)	1.0 ± 0.3 (0.1) *	8.0 (5.0, 12.0)	11.6 ± 8.3 (2.0) *	22.8 ± 15.3 (3.9) *
Rd	RG (14)	1.0 ± 1.1 (0.3)	8.0 (2.0, 24.0)	7.8 ± 13.0 (3.0)	18.2 ± 37.7 (10.1)
	BG (8)	0.9 ± 0.4 (0.2)	8.0 (6.0, 8.0)	3.6 ± 7.1 (1.7)	9.6 ± 15.7 (5.6)
Rg3	RG (19)	1.6 ± 1.0 (0.2)	4.0 (3.0, 5.0)	9.0 ± 5.6 (1.3)	8.7 ± 6.0 (1.4)
	BG (18)	7.3 ± 2.3 (0.5) *	5.0 (2.1, 6.0) *	64.7 ± 23.4 (5.5) *	63.0 ± 27.1 (6.4) *
Rg5	RG (16)	1.4 ± 0.6 (0.2)	5.0 (3.0, 5.0)	3.4 ± 2.7 (0.6)	3.6 ± 2.5 (0.6)
	BG (18)	7.6 ± 2.7 (0.6) *	5.0 (5.0, 5.1)	38.9 ± 13.3 (3.1) *	37.4 ± 12.0 (2.8) *
Rk1	RG (7)	0.7 ± 0.3 (0.1)	5.0 (2.0, 6.0)	0.6 ± 1.3 (0.3)	1.3 ± 1.6 (0.6)
	BG (18)	2.8 ± 1.0 (0.2) *	5.0 (5.0, 5.1)	15.0 ± 5.0 (1.2) *	14.3 ± 5.0 (1.2) *
Rh2	RG (17)	1.6 ± 1.1 (0.3)	10.0 (2.0, 14.0)	9.5 ± 7.3 (1.7)	8.2 ± 5.6 (1.4)
	BG (18)	16.9 ± 12.4 (2.9) *	10.0 (2.0, 14.0)	157.1 ± 107.3 (25.3) *	152.1 ± 111.8 (26.3) *
CK	RG (18)	11.7 ± 6.3 (1.5)	12.0 (8.0, 30.0)	103.5 ± 58.5 (13.4)	136.2 ± 74.8 (17.6)
	BG (18)	11.7 ± 13.5 (3.2)	12.0 (2.0, 24.0)	123.0 ± 135.7 (32.0)	147.5 ± 169.4 (39.9)
PPD	RG (18)	3.8 ± 2.3 (0.5)	24.0 (8.0, 30.0)	37.5 ± 33.5 (7.7)	80.0 ± 71.9 (16.9)
	BG (17)	5.1 ± 3.5 (0.9)	24.0 (12.0, 30.0)	57.8 ± 47.6 (11.2)	107.3 ± 74.5 (18.1)
Re ^2^	RG (0)	–	–	–	–
	BG (0)	–	–	–	–
Rg1 ^3^	RG (0)	–	–	–	–
	BG (0)	–	–	–	–
Rh1 ^4^	RG (0)	–	–	0.0 ± 0.0 (0.0)	–
	BG (16)	1.2 ± 0.6 (0.2)	5.0 (3.0, 10.0)	6.2 ± 6.8 (1.6)	6.3 ± 6.7 (1.7)
PPT	RG (19)	5.8 ± 4.4 (1.0)	14.0 (6.0, 30.0)	56.0 ± 54.4 (12.5)	95.5 ± 86.9 (19.9)
	BG (18)	6.1 ± 4.6 (1.1)	24.0 (5.0, 30.0)	63.5 ± 49.6 (11.7)	102.2 ± 76.2 (18.0)

The data are summarized as mean ± standard deviation (standard error), except for T_max_, which is presented as the median (min, max). TRT, treatment; C_max_, maximum concentration; T_max_, time to reach C_max_; AUC_24_, area under the concentration–time curve from 0 to 24 h; AUC_last_, AUC from 0 to the actual point in time with respect to the last concentration above the lower limit of quantification (LLOQ). ^1^ The observed number of C_max_ and T_max_ and the calculated number of AUC_last_. The AUC_24_ of all ginsenosides was calculated as the number of RG 19 and BG 18. ^2^ All Re concentrations were below the LLOQ. ^3^ All Rg1 concentrations, except for one, were below the LLOQ. ^4^ All RG concentrations were not detected or were below the LLOQ. * Statistically significant at *p* < 0.05.

**Table 2:** Pharmacokinetic (PK) parameters of functional ginsenoside groups in healthy male volunteers after a cross-over administration of black ginseng (BG) and red ginseng (RG).

Ginsenosides	TRT (N ^1^)	PK Parameters			
		C_max_ (ng/mL)	T_max_ (h)	AUC_24_ (ng·h/mL)	AUC_last_ (ng·h/mL)
Rb1 + Rg1 + Rg3	RG (19)	4.0 ± 1.4 (0.3)	6.0 (3.0, 12.0)	60.0 ± 20.9 (4.8)	100.8 ± 37.3 (8.6)
	BG (18)	8.8 ± 2.6 (0.6) *	5.0 (2.1, 6.0) *	93.6 ± 31.2 (7.4) *	118.2 ± 44.3 (10.4)
Rg3 + Rg5 + Rk1	RG (19)	2.8 ± 1.9 (0.4)	5.0 (2.0, 5.0)	12.8 ± 8.7 (2.0)	12.2 ± 8.6 (2.0)
	BG (18)	17.5 ± 5.7 (1.3) *	5.0 (5.0, 5.1)	118.3 ± 37.0 (8.7) *	116.2 ± 40.1 (9.4) *

The data are summarized as mean ± standard deviation (standard error), except for T_max_, which is presented as the median (min, max). TRT, treatment; C_max_, maximum concentration; T_max_, time to reach C_max_; AUC_24_, area under the concentration–time curve from 0 to 24 h; AUC_last_, AUC from 0 to the actual point in time with respect to the last concentration above the LLOQ. ^1^ The observed number of C_max_ and T_max_ and the calculated numbers AUC_24_ and AUC_last_. * Statistically significant at *p* < 0.05.

**Table 3:** Pharmacokinetic (PK) parameters of total ginsenosides in healthy male volunteers after a cross-over administration of black ginseng (BG) and red ginseng (RG).

Ginsenosides	TRT (N ^1^)	PK Parameters			
		C_max_ (ng/mL)	T_max_ (h)	AUC_24_ (ng·h/mL)	AUC_last_ (ng·h/mL)
Total	RG (19)	24.7 ± 6.9 (1.6)	14.0 (5.0, 30.0)	331.6 ± 85.3 (19.6)	547.6 ± 134.3 (30.8)
	BG (18)	45.5 ± 25.4 (6.0) *	9.0 (5.0, 14.0) *	592.4 ± 285.9 (67.4) *	767.0 ± 317.4 (74.8) *

The data are summarized as mean ± standard deviation (standard error), except for T_max_, which is presented as the median (min, max). TRT, treatment; C_max_, maximum concentration; T_max_, time to reach C_max_; AUC_24_, area under the concentration–time curve from 0 to 24 h; AUC_last_, AUC from 0 to the actual point in time with respect to the last concentration above the lower limit of quantification (LLOQ). ^1^ The observed number of C_max_ and T_max_ and the calculated numbers AUC_24_ and AUC_last_. * Statistically significant at *p* < 0.05.

## Data Availability

The individual data in the current study are partially available only upon request from the corresponding authors because the data are strictly confidential, owing to ethical issues.
